# Systematic analysis of palatal transcriptome to identify cleft palate genes within TGFβ3-knockout mice alleles: RNA-Seq analysis of TGFβ3 Mice

**DOI:** 10.1186/1471-2164-14-113

**Published:** 2013-02-20

**Authors:** Ferhat Ozturk, You Li, Xiujuan Zhu, Chittibabu Guda, Ali Nawshad

**Affiliations:** 1 Department of Oral Biology, College of Dentistry, University of Nebraska Medical Center, 40th and Holdrege St, Lincoln, NE 68583, USA; 2 Department of Genetics and Cell Biology, University of Nebraska Medical Center, Omaha, NE, USA; 3 Bioinformatics and Systems Biology Core Facility, University of Nebraska Medical Center, Omaha, NE, USA; 4 Current address: Department of Molecular Biology and Genetics, Canik Basari University Canik, Samsun 55080, Turkey

**Keywords:** RNA-Seq, Next-generation sequencing, TGFβ3, Knockout, Transcriptome, Cleft palate, Palatogenesis, Palate, Craniofacial

## Abstract

**Background:**

In humans, cleft palate (CP) accounts for one of the largest number of birth defects with a complex genetic and environmental etiology. TGFβ3 has been established as an important regulator of palatal fusion in mice and it has been shown that TGFβ3-null mice exhibit CP without any other major deformities. However, the genes that regulate cellular decisions and molecular mechanisms maintained by the TGFβ3 pathway throughout palatogenesis are predominantly unexplored. Our objective in this study was to analyze global transcriptome changes within the palate during different gestational ages within TGFβ3 knockout mice to identify TGFβ3-associated genes previously unknown to be associated with the development of cleft palate. We used deep sequencing technology, RNA-Seq, to analyze the transcriptome of TGFβ3 knockout mice at crucial stages of palatogenesis, including palatal growth (E14.5), adhesion (E15.5), and fusion (E16.5).

**Results:**

The overall transcriptome analysis of TGFβ3 wildtype mice (C57BL/6) reveals that almost 6000 genes were upregulated during the transition from E14.5 to E15.5 and more than 2000 were downregulated from E15.5 to E16.5. Using bioinformatics tools and databases, we identified the most comprehensive list of CP genes (n = 322) in which mutations cause CP either in humans or mice, and analyzed their expression patterns. The expression motifs of CP genes between TGFβ3+/− and TGFβ3−/− were not significantly different from each other, and the expression of the majority of CP genes remained unchanged from E14.5 to E16.5. Using these patterns, we identified 8 unique genes within TGFβ3−/− mice (*Chrng, Foxc2, H19, Kcnj13, Lhx8, Meox2, Shh,* and *Six3)*, which may function as the primary contributors to the development of cleft palate in TGFβ3−/− mice. When the significantly altered CP genes were overlaid with TGFβ signaling, all of these genes followed the Smad-dependent pathway.

**Conclusions:**

Our study represents the first analysis of the palatal transcriptome of the mouse, as well as TGFβ3 knockout mice, using deep sequencing methods. In this study, we characterized the critical regulation of palatal transcripts that may play key regulatory roles through crucial stages of palatal development. We identified potential causative CP genes in a TGFβ3 knockout model, which may lead to a better understanding of the genetic mechanisms of palatogenesis and provide novel potential targets for gene therapy approaches to treat cleft palate.

## Background

Cleft palate (CP) is the second most common birth defect (1/800 live births) in humans, and is caused by the lack of fusion of the embryonic palatal shelves early in gestation (6–10 weeks) [1]. The formation of a continuous palate is a complex process involving multiple steps, including: palatal shelf growth, elevation, attachment, and fusion. The stages of palatogenesis are regulated by numerous genes that are essential for normal palate development. Our laboratory has a long-standing interest in identifying the genetic and molecular mechanisms that regulate palatogenesis in order to understand the factors involved in the development of orofacial clefts [2-11]. It has been shown that both genetic and environmental elements contribute to the development of cleft palate [12,13]. We previously presented that Transforming Growth Factor (TGF)-β isoforms play essential roles in the regulation of palatal morphogenesis, including the finding that TGFβ3 mediates palatal fusion both in primary cells and organ culture [5,14,15]. According to several population-based mutation screening studies, TGFβ3 is considered a candidate gene for non-syndromic CP in humans [16-22]. Furthermore, TGFβ3 knockout mice are born with CP but lack other major defects [23-25].

In our earlier studies [26], we examined gene expression during palatal fusion in normal mice using microarray analysis and detected several genes essential for completion of palatal development. As genome sequencing technologies advanced, it has become feasible to systematically analyze global transcriptomal changes and identify the key molecular components in the developing palate during crucial stages of palatogenesis. Failure to regulate functional or structural genes during these stages may result in cleft palate. Next-generation sequencing (NGS) technologies, or RNA-Seq, have recently emerged as a revolutionary tool of transcriptomics [27] by revealing the complex landscape of the transcriptome with high-throughput at an incomparable level of sensitivity and accuracy [27,28]. The results of RNA-Seq demonstrate high levels of reproducibility for both technical and biological replicates [29,30]. The analysis of TGFβ3 knockout mice by RNA-Seq provides a valuable resource to facilitate our understanding of the complex genetic and molecular mechanisms of palatogenesis. This technology also allows us to detect allelic and splice variants of some of the genes involved in palate development in TGFβ3 knockout mice, which is currently beyond the scope of this article.

Evidence suggests that TGFβ3 is essential to palato-genesis, especially during embryonic days (E) E14.5 to E16.5. However, the cellular mechanisms maintained and regulated by TGFβ3 signaling have not been extensively explored to enable an understanding of those genes functionally regulated by TGFβ3 during normal palatogenesis or those genes deregulated in TGFβ3-null mice. Our objective in this study was to analyze transcriptome changes and their contribution to the development of CP among different gestational ages and TGFβ3 knockout alleles. In this study, we analyzed the transcriptome of TGFβ3 mice by RNA-Seq at crucial stages of palatogenesis, including: palatal convergence, adhesion, and fusion; and we characterized some crucial transcripts that may play key regulatory roles throughout palatal development. We used palatal shelves extracted from E14.5, E15.5 and E16.5 allelic mice to analyze differential expression patterns of their transcriptome throughout the palatal developmental process. The overall transcriptome analysis of TGFβ3 WT mice revealed that almost 6000 genes were upregulated during the transition from E14.5 to E15.5 and more than 2000 were downregulated from E15.5 to E16.5, which suggests that regulation of these transcripts is essential during the stages of palate development. In order to streamline the analysis of the high number of transcripts, we downsized the list of genes to CP genes only. Using biological databases, we categorized a comprehensive inventory of CP genes both in human and mouse genomes and focused on their differential expressions and biological interpretations. Unexpectedly, the expression patterns of CP genes between TGFβ3+/− and TGFβ3−/− were not significantly different from each other, and the expression of the majority of CP genes remained unchanged from E14.5 to E16.5. Using this data, we identified 8 unique genes (*Chrng, Foxc2, H19, Kcnj13, Lhx8, Meox2, Shh,* and *Six3)*, which may regulate cleft palate formation in TGFβ3−/− mice. We used Ingenuity Pathway Analysis (IPA) software to analyze downstream effects of these genes on biological functions, molecular networks, and regulatory pathways- particularly TGFβ signaling. This is the first genome scale analysis of the palatal transcriptome of mice generated by RNA-Seq technology during critical stages of palatogenesis. This data will allow the comprehensive analysis of TGFβ signaling during palatogenesis and provide information on the chronological regulation of downstream TGFβ-activated transcription factors that function to induce cellular differentiation, transformation, and apoptosis.

## Results

### Analysis of RNA-seq data

**Figure 1:**
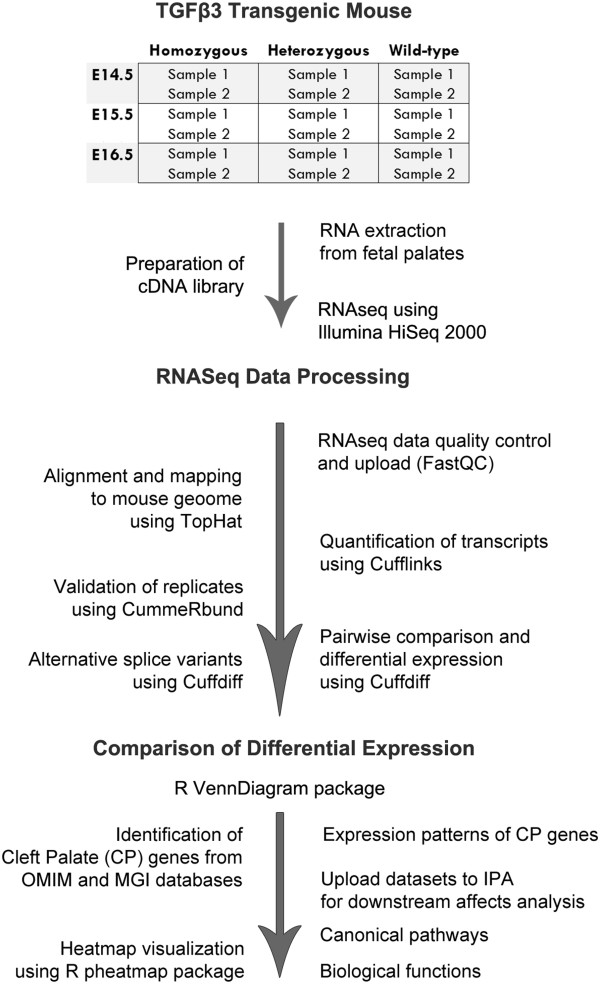
**Experimental workflow of the study.** RNA samples were extracted from mouse palatal tissues of TGFβ3 knockout mice (homozygous, heterozygous, and wildtype) at three developmental stages (E14.5, E15.5, and E16.5); libraries were prepared, converted to cDNA, and sequenced by using the Illumina HiSeq2000 next generation sequencer. Fastq files were uploaded to the server for quality control analysis of sequence reads using FastQC. There were no sequence manipulation processes performed for any fastq file, given that the quality score was high at both the 3^′^ and 5^′^ ends. All of the reads were mapped to the reference genome (mm9, built name NCBIM37) by using TopHat. Quantification of transcripts, statistics tests for differential expression, and detection of splice variants were performed using Cufflinks; quality assessments of biological and technical replicates were performed using CummeRbund; and pairwise comparisons of samples and differential expression of transcripts were analyzed using CuffDiff. Venn diagrams of all significantly altered (FC ≥ 2.0) transcripts were drawn using the R VennDiagram package [75]. CP related genes (n = 322) were extracted from human (OMIM) and mouse (MGI) genome databases. CP genes were classified based on their patterns of expression (n = 9) for each genotype and their heatmaps were generated using the R pheatmap package. Expression pattern groups of CP genes were uploaded to IPA software as datasets and core analyses were run to detect downstream effects: canonical pathways and biological functions relevant to sample datasets.

As outlined in the experimental workflow of our study (Figure 1), RNA samples were extracted from mouse palatal tissues of TGFβ3 knockout mice (homozygous (−/−), heterozygous (+/−), and wildtype (+/+)) at three developmental stages (E14.5, E15.5, and E16.5); libraries were prepared, converted to cDNA, and sequenced by using the Illumina HiSeq2000 next generation sequencer. The bioinformatics analysis of RNA-Seq data was processed using the Tuxedo protocol [31], which includes TopHat, Cufflinks and CummeRbund packages from R. The CP genes were identified from OMIM and MGI databases and downstream effects were analyzed using IPA.

Our data clearly demonstrate that TGFβ3 wildtype (+/+) palates from E15.5 expressed increased TGFβ3 protein, double the expression of heterozygous (+/−) and as expected the homozygous (−/−) palates were completely devoid of any TGFβ3 expression (Additional file 1). Extracted RNA samples were evaluated by RNA integrity numbers (RIN) to monitor quality and degradation with BioAnalyzer (Agilent Technologies, Palo Alto, CA). We confirmed that all of the palatal RNA samples had an RIN ≥ 8.4, indicating the high quality and low degradation of our experimental samples (Additional file 2). The RNA libraries were prepared for cDNA conversion and the samples were sequenced by Illumina techniques using HiSeq 2000. We obtained total reads from the RNA-Seq samples ranging from 14 million to 96 million (Additional file 3). Among all samples, only 4 samples have less than 40 million reads. We did not apply sequence manipulations on either 3^′^ or 5^′^ ends because the RNA-Seq sequencing quality was high across all samples based on our FastQC data quality control analysis. The relative abundance of transcripts was normalized to the total read number and shown as fragments per kilobase per million (FPKM) and mapped via Cufflinks.

**Figure 2:**
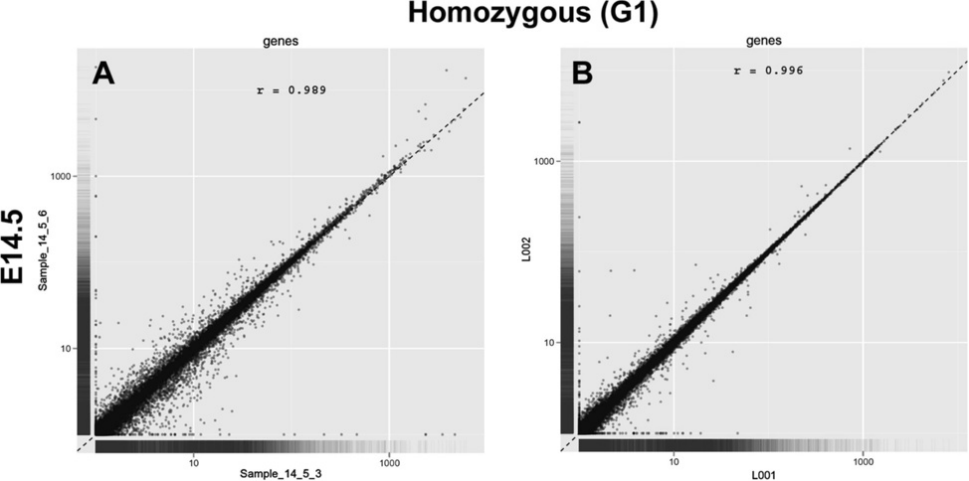
**Validation of biological and technical replicates using CummeRbund.** The read numbers per transcript between the (**A**) biological and (**B**) technical replicates were compared. Since the read number per transcript (FPKM value) ranged from 0 to >10,000, the read number + 1 was transformed by log10. Each dot represents data from one transcript. There was a high correlation between the two sequencing results (r = 0.98, Pearson’s correlation; p < 0.001 two tail). Additional scatter plots for the other samples are shown in the Additional file 4.

In order to evaluate the reproducibility of our experimental samples and procedures, two independent biological and technical replicates for each gestational age and each allele resulting in 36 (n = 9 × 2 × 2) paired-end samples were assayed [32]. Using the scatter plots generated by CummeRbund, biological and technical replicates of our samples were validated based on the comparison of their read numbers per transcript. We detected a high correlation among both the biological and technical replicates of each gestational age and allele (Figure 2) (r = 0.98, Pearson’s correlation; p < 0.001). The replicates of E14.5 of TGFβ3−/− mice are presented in Figure 2. Additional scatter plots for all of the other samples with their replicates are shown in Additional file 4. Because the expression values (FPKM) of certain CP related genes differed significantly among the gestational ages, we transformed the FPKM value of each gene into log_10_ (FPKM + 1), rounded up to 2 decimal places, and used transformed values for further analysis.

### Overall transcriptome expression profile

Using the R VennDiagram package, we created Venn diagrams to illustrate the number of transcripts in each experimental group that passed the significance and fold change (FC ≥ 2.0) thresholds designated by Cufflinks (Additional file 5). From our overall transcriptome analyses of TGFβ3 knockout mice alleles (Table 1), in all genotypic groups, the majority of the differentially expressed transcripts between E14.5 and E15.5 were upregulated: 62% for TGFΒ3−/− (n = 1721), 53% for TGFΒ3+/− (n = 1063), and 80% for WT (n = 5946). Out of 1721 transcripts upregulated in TGFΒ3−/−, 1030 (60%) were shared with WT. Interestingly, a significant portion of the upregulated genes in WT (30%, n = 1792) were downregulated between E15.5 and E16.5, but not in TGFΒ3−/−’s E15.5 to E16.5 (Table 1). The names of the genes significantly increased from E14.5 to E15.5 followed by downregulation in E16.5 and their corresponding Venn Diagrams are presented in Additional file 6. This *first-up-then-down pattern of expression* suggests that these transcripts may be crucial at E15.5, which corresponds to the stage of midline epithelial seam (MES) degradation and palatal fusion. Since it was not feasible to analyze all significantly altered genes during palatal stages, we have shortlisted the genes to be analyzed to CP-causing genes only.

**Table 1:** Number of transcript changes among TGFβ3 alleles

***All Significant***	**E14.5 - E15.5**	**E14.5 - E16.5**	**E15.5 - E16.5**
**TGFβ3−/−**	1806	2096	747
**TGFβ3+/−**	1170	1262	783
**WT**	5966	1903	2256
***Upregulated***			
**TGFβ3−/−**	1721	1941	629
**TGFβ3+/−**	1063	1181	574
**WT**	5945	1835	417
***Downregulated***			
**TGFβ3−/−**	86	155	118
**TGFβ3+/−**	108	81	209
**WT**	21	68	1839
TGFβ3−/−: Homozygous			
TGFβ3+/−: Heterozygous			
WT: Wild-type			

### Identification of CP related genes and their expression patterns

Following the identification of genes causing CP in humans and mice, as described in the methods, we generated a Venn diagram to detect common genes between humans and mice that cause CP after becoming dysfunctional (Additional file 7). We identified 37 common genes that cause CP in both species. Within the human CP list, there were 194 transcripts that share orthologous copies with mice. We approached these genes as putative CP genes, considering that their KO models have not been generated yet. Overall, the CP gene list we developed includes 322 genes: 128 genes derived from mouse and 230 genes derived from human databases (Table 2). For an enhanced visualization of the expression values (FPKM) of all CP genes (n = 322), a Circos image was drawn based on the circular composition of CP genes located on human chromosomes [33] (Additional file 8).

**Table 2:** List of genes causing CP according to MGI and OMIM databases (n = 322)

Acvr1	Lhx8	Cask	ATR	FLNB	MEGF10	RPL11
Akap8	Luzp1	Cdkn1c	ATRX	FLVCR2	MID1	RPL5
Ap2b1	Meox2	Chd7	B3GALTL	FRAS1	MKS1	RPS17
Apaf1	Mmp14	Col2a1	B3GAT3	FREM2	MLL2	RPS19
Bmp7	Mmp16	Dhcr7	B9D1	FTO	MSX2	RPS7
Bnc2	Mn1	Dlx5	BCOR	G6PC3	MTHFR	SALL4
Boc	Mnt	Efnb1	BMP2	GABRB3	MUTYH	SC5DL
Cacna1s	Msc	Eya1	BMP4	GATA6	MYH3	SCARF2
Cdon	Ncoa6	Fgf10	BMPER	GCMB	NBS1	SEMA3E
Chrd	Ncor2	Fgfr1	BRAF	GDF1	NEB	SF3B4
Chuk	Osr2	Fgfr2	BRIP1	GDF6	NEK1	SHFM3
Crebbp	Pax9	Flna	BUB1B	GJA1	NIPBL	SLC26A2
Crk	Pbx1	Foxc2	CANT1	GJB2	NKX2-5	SLC35D1
Ctgf	Pcgf2	Foxe1	CASR	GLI3	NKX2-6	SMOC1
Ctnnb1	Pdgfc	Gad1	CDC6	GPC3	NSD1	SMS
Dlg1	Pdgfra	Hoxa2	CDH1	GUSB	OFC4	SOX2
Dlx1	Pds5a	Impad1	CFC1	H19	OFD1	SPECC1L
Dlx2	Pds5b	Inpp5e	CHRNA1	HCA1	ORC1	SPINT2
Ednrb	Phc1	Irf6	CHRND	HPGD	OTX2	SPTLC1
Ext1	Phc2	Kcnj2	CHRNG	HVEC	PAX3	SRY
Fgf18	Pkdcc	Msx1	CHST14	HYAL1	PAX8	STRA6
Fgf9	Prdm16	Pitx1	CHSY1	HYLS1	PEX7	TAB2
Fign	Prrx2	Prrx1	COL11A1	ICK	PHF8	TBX15
Foxf2	Ptprf	Ror2	COL11A2	IL1B	PIGL	TBX19
Foxg1	Ptprs	Runx2	COL9A2	IL1RN	PIGV	TBX4
Fst	Rad23b	Satb2	COLEC11	IMAGE	PIK3CA	TBX5
Fuz	Rfng	Shh	COMT	IRF1	PLCB4	TCTN2
Fzd1	Rspo2	Six3	CRLF1	KAL1	POLR1C	TGFβ1
Fzd2	Ryk	Sox9	DGCR2	KAT6B	POLR1D	TGFβR1
Gab1	Ryr1	Sumo1	DHCR24	KCNJ13	POMT1	TMCO1
Gli2	Sfn	Tbx1	DHODH	KIAA1289	POMT2	TMEM216
Gpr124	Shox2	Tbx22	DIS3L2	KIF22	PORCN	TMEM67
Grb2	Sim2	Tcof1	DOK7	KIF7	PQBP1	TRIM37
Gsk3b	Skor2	Tfap2a	DXS423E	KLF6	PROKR2	TRPS2
Hand2	Slc32a1	TGFβ3	DYNC2H1	KRAS	PTCH1	TWIST1
Hoxa1	Snai1	TGFβr2	EFTUD2	L1CAM	PTEN	UBB
Hs2st1	Snai2	Tp63	EPHB2	LMNA	PTH	VCAN
Ift172	Sox11	ABCD3	ERBB2	LMX1B	PTPN11	WDR65
Ift88	Sox5	ACAN	ERCC5	LRP4	PXMP3	WNT3
Inhba	Tbx10	ACTB	ESCO2	MADH3	RAI1	WNT5A
Inhbb	Tbx2	ALX1	FAM123B	MAP2K1	RAPSN	WNT7A
Itgav	Tbx3	ALX3	FAM20C	MAP2K2	RB1	WT1
Itgb8	TGFβ2	ARHGAP31	FBN1	MASP1	RBM10	ZEB2
Jag2	Tshz1	ARID1B	FGD1	MBTPS2	RECQL4	ZIC2
Jmjd6	Wdr19	ARNT	FGF8	MCOPS8	RIPK4	ZIC3
Kif3a	Zeb1	ASXL1	FGFR3	MED12	RPGRIP1L	ZMPSTE24

**Figure 3:**
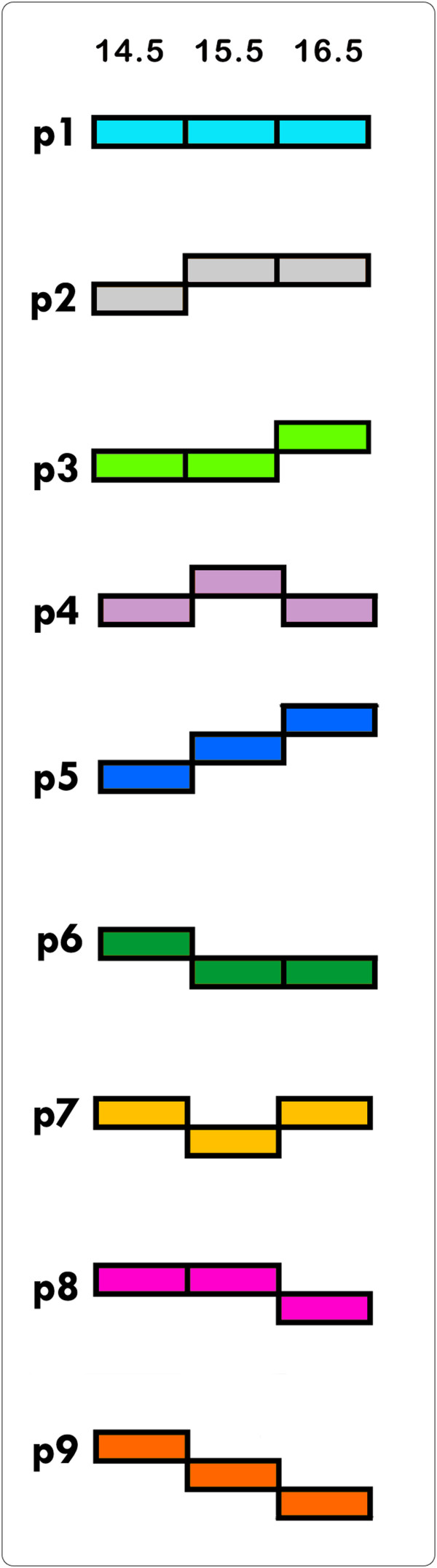
**Expression patterns of CP genes through palatal stages: FPKM values of each CP gene were transformed into log**_**10 **_**(FPKM + 1) and rounded up to 2 decimal places.** These 322 CP genes were categorized into 9 possible expression patterns (p) based on the comparison of the transformed FPKM values with FC ≥ 2.0 across 3 different gestational groups for each genotype (n = 3). The patterns represent the expression value of the transcript *first* from E14.5 to E15.5, *then* from E15.5 to E16.5. Each pattern is color-coded to differentiate patterns for further analysis. **p1** unchanged-unchanged (remain unchanged); **p2** upregulated-unchanged; **p3** unchanged-upregulated; **p4** upregulated-downregulated; **p5** upregulated-upregulated; **p6** downregulated-unchanged; **p7** downregulated-upregulated; **p8** unchanged-downregulated; and **p9** downregulated-downregulated.

On the basis of our differential expression profile data between genotypes and gestational ages (Table 1), we decided to group and analyze the potential CP genes based on their *expression patterns* throughout different stages of palatogenesis. We categorized the 322 CP genes identified above into 9 possible *expression patterns* based on the comparison of their expression values (upregulated, downregulated, or unchanged) across the 3 different gestational age groups analyzed by RNA-seq (E14.5, E15.5, and E16.5) (Figure 3). The patterns (p) represent the change in expression value of the transcript *first* from E14.5 to E15.5, and *second* from E15.5 to E16.5, which is explained in detail in Figure 3. The genes in each *expression pattern group* are listed and common genes among them are diagrammed in Additional file 9.

**Figure 4:**
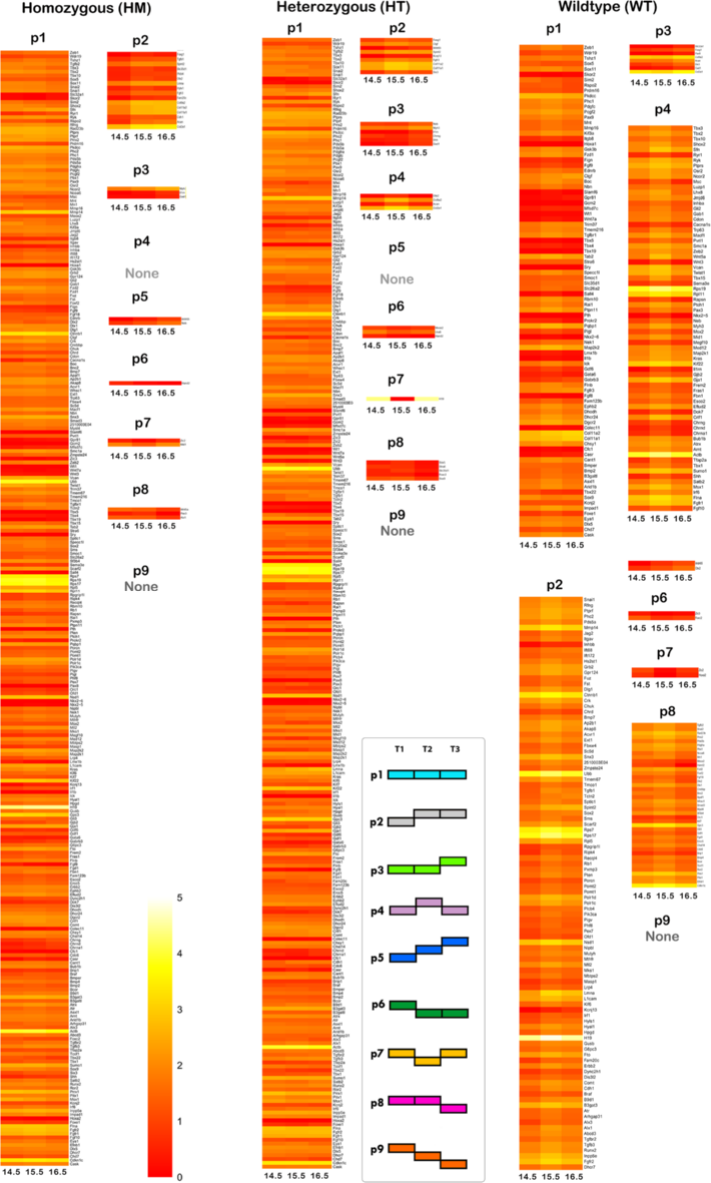
**CP genes represented in pattern-oriented heatmaps.** Transcripts were clustered based on expression patterns (n = 9) and heatmaps were constructed using the R pheatmap package. Each column corresponds to the transformed FPKM value of transcripts in duplicate, and each row corresponds to a gene. None of the genotypes had transcripts clustered in p9. Additionally, p4 in TGFΒ3−/− and p5 in TGFΒ3+/− did not contain transcripts. Therefore, the total number of CP gene patterns/genotype groups is 22. The increasing intensity of red to yellow signifies a higher level of expression (FPKM) in the given sample of a specific transcript. The list of genes for each particular pattern and genotype are shown in Additional file 9.

Considering the differential expression of CP genes throughout palatogenesis, we generated individual heatmaps according to the expression patterns of CP related genes and grouped them into corresponding genotypes (Figure 4). In TGFΒ3−/−, the majority of transcripts (n = 294; 92%) were placed in p1, which denotes unchanged expression of genes throughout gestation. Similarly, in TGFΒ3+/− samples, the majority of transcripts were in p1 (n = 293; 91%). Surprisingly, the list of genes in p1 from TGFΒ3−/− and TGFΒ3+/− were highly comparable (92%) (Additional file 9). The genes clustered in p1 *unique to TGFΒ3−/−,* but not in TGFΒ3+/−*,* were *Chrng, Foxc2, H19, Kcnj13, Lhx8, Meox2, Shh,* and *Six3* (Table 3). We concluded that these 8 genes demand additional attention due to their unchanged level of expression during crucial stages of palatogenesis.

**Table 3:** Unique TGFβ3−/− genes following the p1 pattern

	**TGFβ3−/−**	**TGFβ3 +/−**	**WT**	**Name**	**Type**
**Chrng**	p1	p3	p4	Cholinergic receptor, nicotinic, gamma (muscle)	Transmembrane glycoprotein
**Foxc2**	p1	p8	p6	Forkhead box C2	Transcription factor
**H19**	p1	p7	p2	Imprinted maternally expressed transcript H19	Non-protein coding RNA
**Kcnj13**	p1	p2	p2	Potassium inwardly-rectifying channel, subfamily J, member 13	Inwardly rectifying potassium channel family of proteins
**Lhx8**	p1	p6	p4	LIM homeobox 8	Transcription factor
**Meox2**	p1	p6	p8	Mesenchyme homeobox 2	Antennapedia-like homeobox-containing genes
**Shh**	p1	p3	p4	Sonic hedgehog	Morphogen – Secreted protein
**Six3**	p1	p2	p3	SIX homeobox 3	Transcription factor

Finally, in WT samples, the transcripts were more equally dispersed into p1, p2 and p4 as 29%, 32%, and 23% respectively. These results suggest that in normal mice, the majority of CP transcripts are required to be upregulated between E14.5 and E15.5 (denoted as p2 and p4), which corresponds to the most crucial stages of palatogenesis- the adhesion and the fusion stages [34]. Some of these upregulated genes were eventually downregulated (p4) or remained at the same expression levels (p2), which suggest that they may contribute to the final phase of palate development.

### Downstream pathway analysis of CP genes

**Figure 5:**
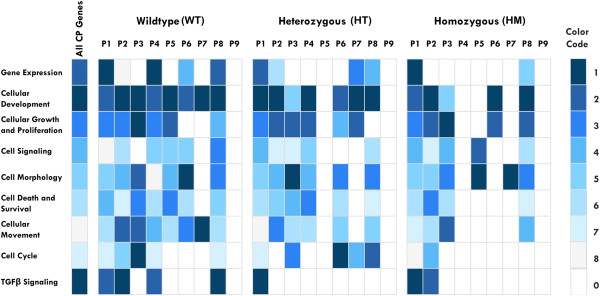
**Downstream effects analysis of CP gene patterns using IPA.** The effects of TGFβ3 alleles on CP genes during palatogenesis were examined in the context of differentially expressed human and mouse genes using Ingenuity Pathway Analysis (IPA). Entrez IDs of each CP gene pattern/genotype group (n = 22) were uploaded to IPA as individual datasets. Each identifier was mapped to its corresponding gene object in the Ingenuity Knowledge Base (IKB). Interactions were then queried between these datasets and all other gene objects stored within IPA to generate a set of overlapping direct interaction networks. The significant genes were categorized, compared to functional categories in the IPA database, and ranked according to their p-values. The cellular processes that are most significantly altered in our datasets were: gene expression, cellular development, cellular growth and proliferation, cell signaling, cell morphology, cell death and survival, cellular movement, and cell cycle. The pattern-oriented distribution of the top cellular biofunctions were ranked and illustrated in a color coded matrix: the darker color represents greater relevance between cellular function and CP gene datasets.

We used the IPA software to determine how the expression pattern-clustered CP genes interconnected with each other to facilitate cellular biofunctions and signaling. According to the core analysis of all CP genes (n = 322) using the IPA, the most significant top 5 cellular functions were *cellular development*, *gene expression*, *cellular growth and proliferation*, *cell signaling* and *cell morphology* (Table 4). Although the numbers of molecules were higher in some cellular functions, the ranking was based on p-values calculated by IPA according to relevance of datasets with IKB. Differentially expressed molecules clustered into GO of these cellular biofunctions are tabulated in Additional file 10. The pattern-oriented distribution of the top cellular biofunctions (n = 8) in relation to the TGFβ pathway are illustrated with a color coded matrix with the darker color representing higher relevance between cellular functions and uploaded CP gene pattern datasets (Figure 5).

**Table 4:** Differentially expressed molecules clustered into GO of cellular biofunctions

**Category**	**p-Value**	**# Mols**
Gene Expression	1.23E-30	151
Cellular Growth and Proliferation	1.48E-23	199
Cell Cycle	2.58E-14	76
Cellular Movement	2.74E-12	107
Cell Signaling	3.20E-22	25
Cell Death	3.44E-12	168
Cell Morphology	4.03E-16	103
TGFβ Signaling	5.02E-18	24

**Figure 6:**
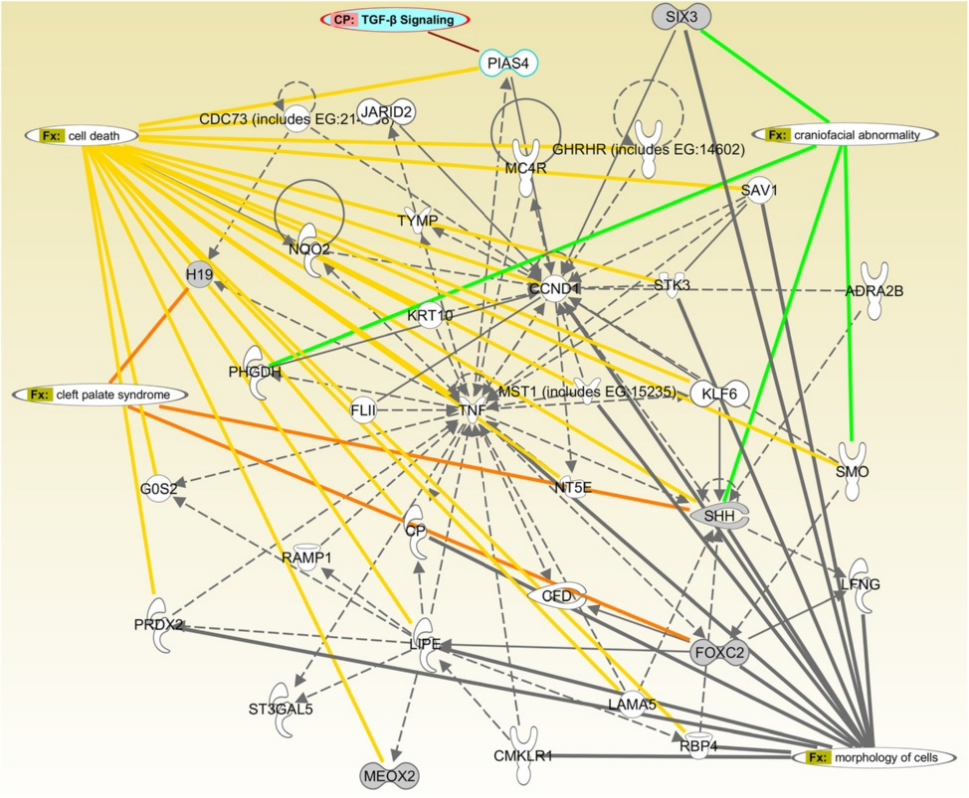
**Cellular biofunction and pathway analysis of CP genes unique to p1 of TGFΒ3−/− (n = 8).** Entrez IDs of CP genes unique to p1 of TGFΒ3−/− were identified using the Venn diagram and uploaded to IPA as an individual dataset. Core analysis was run by default settings on IPA, and the top cellular biofunctions, diseases, and canonical pathways were detected. IPA Path Designer was used to generate the network of these genes through an overlay of the biofunctions and pathways. Genes belonging to the Unique TGFΒ3−/− p1 list were merged into a network (*Foxc2, H19, Meox2, Six3, Shh*) and gray-shaded. Relationships are shown with colored arrows for easy gene identification.

The CP genes unique to p1 of TGFΒ3−/− (n = 8) may have necessary functions for the complete development of palatal shelves. Therefore, we analyzed the interaction of these genes with each other, as well as with key biofunctions and TGFβ signaling using IPA (Figure 6). We found that 5 of these genes (*Shh, Foxc2, Six3, Meox2,* and *H19*) are closely related to each other, with *Tnf* (tumor necrosis factor) and *Ccnd1* (Cyclin d1) being their intersecting molecules. These genes were mostly associated with cell morphology and cell death biofunctions, and were indirectly associated with TGFβ signaling through *Pias4* (protein inhibitor of activated STAT, 4).

**Figure 7:**
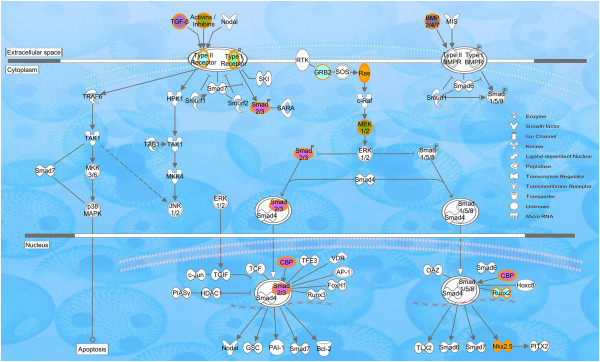
**Characterization of CP genes in relation to the canonical TGFβ signaling pathway.** The cellular localization of CP genes which function in the canonical TGFβ pathway was illustrated using IPA Path Designer. CP gene datasets uploaded to IPA were analyzed using default settings for the core analysis. A total of 19 CP transcripts from these datasets were associated with the TGFβ canonical pathway: ACVR1, BMP2, BMP4, BMP7, CREBBP, GRB2, INHBA, INHBB, KRAS, MAP2K1, MAP2K2, NKX2-5, RUNX2, SMAD3, TGFβ2, TGFβ3, TGFβR1, and TGFβR2. These datasets (n = 7) were selected and overlaid with each other using the IPA Path Designer. Color codes reflect the genotype and expression pattern of CP genes as follows: Orange lines: both TGFβ3−/−p1 and TGFΒβ3+/−p1; green filled: WTp1; light blue filled: WTp2; orange filled: WTp4; and purple filled: WTp8. The types of molecules are annotated in the legend.

According to IPA core analysis, there were 19 molecules associated with TGFβ signaling [Ratio: 19/89 (21%)] among all of the CP genes (n = 322). TGFβ signaling molecules along the pathway and their cellular locations are illustrated in Figure 7. TGFβ signaling transcripts expressed in both TGFΒ3−/− and TGFΒ3+/− were (n = 18): *Acvr1, Bmp2, Bmp4, Bmp7, Crebbp, Grb2, Inhba, Inhbb, Kras, Map2k1, Map2k2, Nkx2-5, Runx2, Smad3, TGFβ2, TGFβ3, TGFβr1, TGFβr2 *(Table 3). All of these molecules followed the p1 expression pattern, which represents a uniform level of expression throughout all time points. However, within WT transcripts, TGFβ-associated molecules were almost equally distributed among p1, p2, p4, and p8. All represented TGFβ pathway molecules (n = 19) were grouped into genotypes according to their expression patterns (Table 5). When the significantly altered CP genes were overlaid with TGFβ signaling, all of these genes followed the Smad-dependent pathway (Figure 7).

**Table 5:** TGFβ pathway molecules grouped into genotypes with patterns

**TGFΒ3−/−**		**TGFΒ3+/−**	**WT**			
**p1**	**p2**	**p1**	**p1**	**p2**	**p4**	**p8**
Acvr1	Tgfβ1	Acvr1	Bmp2	Acvr1	Inhba	Bmp4
Bmp2		Bmp2	Map2k2	Bmp7	Kras	Crebbp
Bmp4		Bmp4	TGFβr1	Grb2	Map2k1	Smad3
Bmp7		Bmp7		Inhbb	Nkx2-5	Tgfβ2
Crebbp		Crebbp		Runx2		
Grb2		Grb2		Tgfβ1		
Inhba		Inhba		Tgfβ3		
Inhbb		Inhbb		TGFβr2		
Kras		Kras				
Map2k1		Map2k1				
Map2k2		Map2k2				
Nkx2-5		Nkx2-5				
Runx2		Runx2				
Smad3		Smad3				
Tgfβ2		Tgfβ1				
Tgfβ3		Tgfβ2				
TGFβr1		Tgfβ3				
TGFβr2		TGFβr1				
		TGFβr2				

## Discussion

### Global transcriptome of TGFβ3 alleles

Palatogenesis is a complex process involving multiple steps, including: palatal shelf growth, elevation, adhesion and fusion (Additional file 11). It requires the orchestration of several key cellular functions (such as cellular proliferation, differentiation, movement, communication, and death) regulated by numerous genes and signaling pathways. It has been well documented that disruption of structural and regulatory genes involved in any stage of palatogenesis, which occurs between E11.5 and E16.5 in mice, contributes to the formation of cleft palate [34]. On the basis of our studies and others [5,6,35], the stages from E14.5 to E16.5 have been identified as the most critical phases. During these stages, the palatal shelves adhere to each other with a seam and the seam begins to disintegrate in order to complete fusion, resulting in a confluent palate. Therefore, we anticipated that the majority of the genes contributing to physiological palatogenesis would be progressively regulated during these phases. In this study, we took advantage of high-throughput sequencing technology, RNA-Seq, to quantitatively analyze the expression of transcripts in mouse embryonic palatal tissues at three developmental stages.

The integrity and quality of the isolated RNA are the primary factors contributing to successful RNA-Seq studies [36], therefore we ensured that our palatal RNA samples were of high quality based on their RIN (>8.4). In our study, we used the “paired end” approach for RNA-Seq, wherein a single 100bp-long read is sequenced at both ends, which allows the tracking of alternate splice junctions, as well as further analysis of SNPs, insertions and deletions; thus providing more accurate and informative sequencing results from our samples [37,38]. We submitted the raw reads of RNA-Seq data (fastq files) to SRA and the assembly data to the Transcriptome Shotgun Assembly Database.

Our global transcriptome analyses of TGFβ3 alleles showed that significant numbers of transcripts (~6000 genes) were differentially expressed between E14.5 and E15.5, particularly within the WT fetuses, indicating functional roles of these genes during this time period of palatogenesis (Table 1). Interestingly, we found lower numbers of transcriptome alterations between the same time points within the TGFβ3−/− mice (n = 2770), which can be explained by the consequential suppression of gene clusters due to inactivation of the TGFβ3 gene. However it was surprising to observe that the lowest number of transcriptome changes were in TGFβ3+/− mice, n = 1993, which are phenotypically identical to WT mice [25]. This may suggest that only this number of gene alterations is sufficient to enhance the palatal growth and fusion.

Furthermore, in normal mice (WT) the majority of transcripts were upregulated during the early transition of palatogenesis (E14.5 - E15.5) and downregulated during the later transitions (E15.5 - E16.5), suggesting that these are critical stages of palatogenesis requiring extensive gene regulation. This pattern of upregulated-downregulated expression suggests that particular CP genes may follow the same expression pattern to stimulate disintegration of the palatal seam, resulting in a confluent palate.

### Understanding the expression of CP genes

The OMIM and MGI databases provide up-to-date and adequately classified information about genetic diseases in humans and mice, respectively; therefore, the identification of CP related genes from either species was uncomplicated. However the merger of the information from these databases required further computer analysis to identify common and individual CP genes between the two species (Additional file 7). To our knowledge, our list of CP genes (Table 2) covers the most comprehensive number of genes (n = 322), which cause CP when mutated in either species alone or both species [39,40].

It was expected that TGFβ3 allelic mice follow 9 different expression pattern combinations during the E14.5-E15.5 and the E15.5-E16.5 transitions (Figure 3). Interestingly, none of the genes followed pattern 9, which represents the continuous downregulation of the transcript throughout all palatal stages. We observed that the majority of the CP genes were equally clustered into p2, p4, and p8 in WT samples (WT), representing normal palate development (Figure 4). Conversely, in both the TGFΒ3−/− and TGFΒ3+/− samples, almost all CP transcripts (91%) were accumulated in p1, and demonstrated high similarity between datasets (92%) (Additional file 9). This suggests that expression regulation of CP genes in both of these allelic mice was either delayed or abandoned.

### Unique TGFΒ3−/− genes following the p1 pattern

Although the pattern of transcriptional expression between TGFΒ3−/− and TGFΒ3+/− are unexpectedly similar, phenotypic expression of CP occurs only in TGFΒ3−/− and not in TGFΒ3+/− pups [24,25]. This suggests that the unique p1 genes of TGFΒ3−/− (n = 8; *Chrng, Foxc2, H19, Kcnj13, Lhx8, Meox2, Shh,* and *Six3*) may play crucial roles in the completion of palatogenesis. Therefore, we analyzed their specific functions within the cell, their roles in palatogenesis, and their interactions with the TGFβ signaling pathway using IPA and GeneCards [41].

Chrng (Cholinergic receptor, nicotinic, gamma) is a transmembrane glycoprotein and has been shown to play a role in neuromuscular organogenesis and ligand binding [42]. Mutation of Chrng causes the developmental disorder multiple pterygium syndrome in humans, exhibited by isolated CP, short stature, vertebral (spine) defects, joint contractures, and webbing of the neck, armpit, elbow, and knee [42,43]. Pterygium is also associated with Irf6 and p63 mutations in humans [44], and both Irf6 and p63 are known to be closely associated with TGFβ signaling. Nevertheless, according to current literature, we could not find any relationships between Chrng and other molecules of the TGFΒ3−/− unique p1 list (Figure 6), or the TGFβ pathway.

Foxc2 (Forkhead box C2) is a member of the forkhead box (FOX) family of transcription factors and has been shown to be functional during development of mesenchymal tissues [45]. Mutations in Foxc2 are responsible for the hereditary lymphedema-distichiasis syndrome with CP observed in some patients [46]. In addition, Foxc2 is also involved in cancer metastases. In particular, expression of Foxc2 is induced when epithelial cells undergo epithelial-mesenchymal transition (EMT) [47]. Recently, Lindley at al. (2010) showed that Foxc2 may play a role in EMT maintenance when human mammary epithelial cells are treated with TGFβ [45]. (The Foxc2 knockout mouse is not available, according to KOMP). Overall, these relationships suggest that Foxc2 could be a vital element of palatal confluency through facilitation of EMT. Furthermore, failure of proper Foxc2 transcript regulation may result in CP in TGFβ3−/− fetus.

H19 is an imprinted maternally expressed transcript and expresses a long noncoding RNA. It has been shown that an enhancer deletion affects the expression of both H19 and Insulin-like growth factor 2 (Igf2), thus the Igf2 and the H19 genes are proposed to utilize a set of common enhancers [48,49]. When Thomas et al. (1997) produced transgenic mice that express the Igf2 gene under the control of the H19 enhancers, a large fraction of homozygous mice developed CP [44]. In our network analysis (Figure 6), we found that H19 is indirectly related with TGFβ signaling through TNF, which suggests that it may play a crucial role during palatogenesis under regulation of TGFβ.

The Kcnj13 gene encodes a member of the inwardly rectifying potassium channel family of proteins and regulates ion transmembrane transport and mutations in Kcnj1 are associated with snowflake vitreoretinal degeneration [50]. There were no significant relationships found between Kcnj12 and other molecules of the TGFΒ3−/− unique p1 list (Figure 6), or the TGFβ pathway. However, this does not rule out its potential regulatory interaction with TGFβ signaling, and therefore demands additional studies to determine its role during palate development.

Lhx8 is a member of the LIM homeobox family of transcription factor proteins, which are involved in patterning and differentiation of various tissue types. Lhx8 is expressed in the mesenchyme of the mouse palatal structures throughout their development [11]. Zhao et al. (1999) showed that in Lhx8 homozygous mutant embryos, the bilateral primordial palatal shelves formed and elevated normally, but they often failed to make contact and to fuse properly, resulting in a cleft secondary palate [51]. Inoue M et al. (2006) proposed that the L3/Lhx8 gene contributes to epithelial mesenchymal interactions during facial morphogenesis and that Fgf-8b and Tgfβ3 were responsible for Lhx8 expression in the maxillary process [52]. Our RNA-Seq study of TGFβ3 −/− fetuses further confirmed the requirement of upregulated expression of Lhx8 throughout palatal morphogenesis.

The Meox2 gene (mesenchyme homeobox 2) encodes a member of a subfamily of non-clustered, diverged, antennapedia-like homeobox-containing genes [53,54]. Jin et al. (2006) found that Meox2 is highly expressed in the posterior region of the developing palate, and the reduction of Meox2 gene levels increased the susceptibility of mice to cleft palate through a novel post-fusion mechanism [53]. The posterior cleft in E15.5 Meox2 −/− mice contained no epithelial cells, therefore they proposed that Meox2 may function to strengthen the fusing zone of palatal shelves. The lack of strong fusion of epithelial cells during stable expression of Meox2 may explain why the palatal shelves of TGFβ3−/− mice attach to each other, but fail to fuse [25]. Recently, Valcourt et al. (2007) showed that ectopic Meox2 suppressed epithelial cell proliferation in cooperation with Tgfβ1, and mediated induction of the cell cycle inhibitor gene p21 [54]. Furthermore, they showed that Meox2 failed to promote EMT and partially blocked Tgfβ1-induced EMT [54]. Considering the requirement of EMT during palatal fusion, Meox2 expression needs to be downregulated at the later stages of palatogenesis, as observed in both WT and TGFβ3+/− mice in our study.

Sonic hedgehog, Shh, is a member of the hedgehog family of secreted proteins and has been implicated as the key inductive morphogen in patterning of vertebrates during organogenesis; particularly the ventral neural tube, the anterior-posterior limb axis, and the ventral somites [55]. Shh signaling plays essential roles in craniofacial development by regulating a number of transcription factors and signaling interactions that take place between the epithelium and mesenchyme during normal palatogenesis [56]. Lan et al. (2009) showed that Shh is predominantly expressed in palatal epithelia and signals directly to the palatal mesenchyme to regulate palatal mesenchyme cell proliferation through maintenance of cyclin D1 and D2 (Ccnd1 and Ccnd2) expression [56]. Recently, Sasaki et al. (2007) showed that Shh expression in the palates of Tgfβ3-null mice was reduced throughout E12.5–E15.5, and thereby proposed that Shh may be involved in TGFβ3 regulation of normal palatal fusion [57]. Our results further support that upregulated expression of Shh is necessary for the successful completion of palatogenesis, as observed in the TGFβ3+/− and WT mice, but not in TGFβ3−/−.

SIX homeobox 3 (Six3) is a transcription factor crucial to embryonic development by providing the necessary instructions for the formation of the forebrain and eye development [58]. Similar to Shh [59], mutations in the homeodomain of the human Six3 gene cause holoprosencephaly and are associated with midline facial cleft - tessier cleft 14 [60]. In mice, it has been shown that Six3 protein increases expression of Ccnd1 protein [61], which is directly related to TGFβ signaling (Figure 6). Our results indicate that Six3 expression has to be upregulated after E14.5 for the precise progression of palate development. Therefore, the interaction of Six3 with TGFβ3 and their mechanism of activation deserve further analysis using biological techniques.

### Cellular biofunctions and TGFβ pathway molecules

The expression pattern-clustered CP genes interconnect each other to facilitate cellular biofunctions and canonical pathways. The relevance of these groups of gene datasets was detected using IPA core analysis (Figure 5).

Our primary objective in this study was to analyze the transcriptomal differences among the alleles of TGFβ3 knockout mice. Thus, it was critical to find the relationship of CP genes with the TGFβ signaling pathway, which encompasses 89 molecules (Figure 7). TGFβ3 controls the fusion of palatal shelves by EMT and apoptosis [5,14,62]. Furthermore, TGFβ1 and TGFβ2 regulate mesenchymal cell proliferation and extracellular matrix synthesis in the palate [63]. Although not all of the molecules of the TGFβ signaling pathway are associated with CP genes, it is noteworthy that all of the related CP genes followed the Smad-dependent pathway. This may indicate that TGFβ3 regulates the CP genes through Smad-signaling, with ancillary involvement of ERK, PI3K, JNK, MAPK, etc. Thus, the promoter regions of these CP genes should be analyzed for Smad-binding elements using ChIP-PCR or ChIP-Seq. The majority of these CP genes followed the p8 expression pattern in WT mice, which represents the unchanged-downregulated expression pattern. This suggests that TGFβ molecules are required during E14.5 and E15.5, but are less necessary during E16.5, which correlates with our previous findings demonstrating the sequential function of TGFβ3 throughout the degradation of the palatal seam [5].

Overall, these sequencing and bioinformatics analysis studies are used as gene identification tools for future experiments to further understand the detailed mechanism of palate development. The 8 putative CP causing genes and Smad-dependent TGFβ signaling molecules will be analyzed in detail using gene and protein expression studies, as well transcription factor and promoter binding assays.

## Conclusions

As a result of the complexity of TGFβ signaling, apparently simple but highly important questions regarding the development of cleft palate remain unanswered, including: what is the genetic footprint of TGFβ3 in normal palatogenesis and how does the deregulation of TGFβ3 signaling cause cleft palate? In this study, we have attempted to answer these questions through the extensive quantitative transcriptome analysis of the palates of TGFβ3 knockout mice through RNA sequencing technology. Our study represents the first detailed analysis of palatal transcriptomes generated by RNA-Seq technology during critical stages of palatogenesis. The dataset described here will provide an enriched resource for searching transcripts that may play key regulatory roles at different stages of palatal development. Furthermore, we identified 8 key genes which may play fundamental roles in the development of cleft palate in TGFβ3−/− mice. Our combined results provide an initial understanding of the complex genetic mechanism of TGFβ3-mediated palatogenesis, and may lead to the identification of genes for the targeted prenatal treatment of cleft palate.

## Methods

### Mouse breeding and genotyping

The generation and genotyping of TGFβ3−/− mice have been described previously [25]. TGFβ3 heterozygous (+/−) C57BL/6J male and female mice were provided by Tom Doetschman (BIO5 Institute, University of Arizona, AZ). Mice were housed in accredited animal facilities at the University of Nebraska Medical Center (UNMC). All procedures were approved by the IACUC (Institutional Animal Care and Use Committee) of UNMC. Homozygous (−/−) embryos were obtained by mating TGFβ3^+/−^ male and female mice. The morning that the vaginal plug was pulled was considered embryonic day (E) 0.5. Tissue samples were individually labeled and embryonic tails were used for genotyping by PCR. Genomic DNA was prepared using the Wizard Genomic DNA Purification Kit (Promega – A1120). The primers used for TGFβ3 were as follows:

TGFβ3 Forward 5^′^ TGGGAGTCATGGCTGTAACT 3^′^

TGFβ3 Reverse 5^′^ CACTCACACTGGCAAGTAGT 3^′^

pMC1-Neo 5^′^ GCCGAGAAAGTATCCATCAT 3^′^

These primers amplified 600 bp and 400 bp fragments for the mutated and WT alleles, respectively. PCR conditions were 30 cycles of 95°C for 30 seconds, 57°C for 45 seconds, 72°C for 1 minute, followed by one cycle of 72°C for 10 minutes.

Typically, 8 fetuses were obtained from each pregnancy in a Mendelian-fashion *3 allelic-genotype* (1:2:1) as 2 TGFβ3−/−, 4 TGFβ3+/−, and 2 WT. Palates were extracted from fetuses as described earlier [2] at *3 time points* (E14.5, E15.5, and E16.5) as *duplicates* and the tissues were stored in RNAlater RNA Stabilization Reagent (QIAGEN, CA) to preserve transcript integrity. In order to evaluate the reproducibility of our experimental samples and procedures, two independent biological and technical replicates were assayed for each gestational age and each allele resulting in 36 (n = 3 × 3 × 2 × 2) samples for sequencing.

### Protein, RNA extraction, construction of small RNA libraries, and RNA-Seq

Total proteins were extracted from the palatal shelves collected from embryonic (E) 15.5 and western blot was performed as previously done in our lab and described in [9,10] using antibody against TGFβ3 (R & D Systems, MN). We extracted the RNA using the RNeasy Kit (QIAGEN, CA) and evaluated the RNA for purity and concentration by ultraviolet spectroscopy (NanoDrop, Wilmington, DE). RNA integrity numbers (RIN) [64,65], based on an algorithm for judging the integrity of RNA samples, were evaluated using the Agilent 2100 Bioanalyzer (Agilent Technologies, Palo Alto, CA). Libraries were prepared using the Illumina mRNA-Seq Sample Preparation Kit (San Diego, CA) according to the manufacturer’s instructions. Briefly, poly(A) + RNA was recovered from 1 μg of total RNA using two rounds of isolation with oligo-dT-coated Sera-Mag magnetic beads. The recovered poly (A) + RNA was then chemically fragmented. RNA fragments were converted to cDNA using SuperScript II and random primers. The second strands were synthesized using RNaseH and DNA Pol I. The ends of the cDNA were repaired using T4 DNA polymerase, T4 polynucleotide kinase, and Klenow DNA polymerase. A single adenosine was added to the 3^′^ end using Klenow fragment (3^′^ to 5^′^ exo minus). Adaptors were attached to both ends of the cDNA using T4 DNA ligase. RNA fragments were extracted from a 2% low range ultra agarose sizing gel. The fragments were amplified by 15 cycles of PCR using Phusion DNA polymerase. Libraries were validated with Agilent Bioanalyzer (Palo Alto, CA). Libraries were diluted to 10 pM and applied to an Illumina flow cell using the Illumina Cluster Station. Sequencing was performed on Illumina HiSeq 2000 according to the manufacturer’s instructions [66,67].

### Mapping and quantification of transcripts

RNA-Seq datasets were analyzed by following the recently published RNA-Seq protocol [31]. The 36 samples (paired-end reads, including biological replicates and technical replicates; n = 3 × 3 × 2 × 2) were individually mapped onto the mouse genome (mm9, build name NCBIM37) by using TopHat v1.4.1 [68-70]. Preprocessing steps were not applied to the raw data (fastq) files because the sequencing quality was very high in both 3^′^ and 5^′^ ends according to the FastQC reports [71]. The relative abundance of reads was normalized to the total read number and shown as fragments per kilobase per million mapped (FPKM) [71]. After the alignment, Cufflinks v1.3.0 [72-74] was used to estimate the abundances of transcripts. Cuffdiff from Cufflinks repository was used to perform pairwise comparisons between different genotypes and gestational groups. The dispersion between each biological replicate was also tested by Cuffdiff, as well as the dispersion between each technical replicate. CummeRbund ( http://compbio.mit.edu/cummeRbund/) software [31] provides functions for creating commonly used expression plots, such as: volcano, scatter and box plots, using processed data from Cuffdiff. We used CummeRbund to generate scatter plots to use for quality assessments of both biological and technical replicates.

We used the VennDiagram [75], an R package that enables the automated generation of highly-customizable, high-resolution Venn diagrams, to illustrate the number of transcripts in each experimental group which passed the significance and fold change (FC ≥ 2.0) filtrations through Cufflinks. Pattern-oriented listings of CP genes were illustrated with GeneVenn, a web application for comparing gene lists (Additional file 5).

### Identification of cleft palate genes

We used the OMIM (Online Mendelian Inheritance in Man) database to retrieve 280 genes and loci involved in genetic diseases associated with CP syndromes in humans. We used information from developmental biology studies demonstrating CP in knockout mouse models and the Mouse Genome Informatics (MGI) website with the mammalian phenotype browser, the international database resource for the laboratory mice, to retrieve 128 genes (out of 223 genotypes) causing CP in mice. The pattern-oriented heatmaps of CP genes were illustrated by using R pheatmap package

### Biological interpretation and pathway analysis

The effects of TGFβ alleles on CP genes during palatogenesis were examined in the context of differentially expressed human and mouse genes using Ingenuity Pathway Analysis (IPA), (Ingenuity Systems, CA), a web-delivered application used to discover, visualize, and explore relevant networks. After performing statistical analysis and filtering of the RNA-Seq data, the Entrez IDs and FPKM values of each group were uploaded to IPA as a dataset. Each identifier was mapped to its corresponding gene object in the IKB. Interactions were then queried between these datasets and all other gene objects stored within IPA to generate a set of direct interaction networks that were overlapped. The significant genes were categorized, compared to functional categories in the IPA database, and ranked according to their p-values. P-values less than 0.05 indicate a statistically significant, nonrandom association between a set of significant genes and a set of all genes related to a given function in the IKB [76]. Through the assessment of differentially expressed genes, cellular processes that were most significantly altered in our dataset were: gene expression, cellular development, cellular growth and proliferation, cell signaling, cell morphology, cell death and survival, cellular movement, and cell cycle. Based on these IPA analyses, the cellular biofunction categories mentioned above and the TGFβ pathway were filtered among other cellular processes and canonical pathways; then ranked according to their p-values and represented as a color coded matrix. Datasets associated with the TGFβ pathway were overlaid using the IPA Pathway Designer.

## Abbreviations

TGFβ3: Transforming growth factor β 3; NGS: Next generation sequencing; RNA-Seq: RNA Sequencing; FC: Fold change; IPA: Ingenuity pathway analysis; E: Embryonic day; CP: Cleft palate; TGFΒ3−/−: Homozygous; WT: Wild-type; TGFΒ3+/−: Heterozygous; FPKM: Fragments per kilo base per million mapped.

## Competing interests

The authors declare that they have no competing interests.

## Authors’ contributions

FO carried out the study design, animal breeding, sample collection, RNA extraction, identification of CP genes, IPA analyses, and drafted the manuscript. YL undertook RNA-Seq data quality control, processing, alignment, visualization, bioinformatics analyses, and helped to draft the manuscript. XZ performed animal breeding, collected palatal tissues and extract RNA CG and AN supervised the design and coordination of the study, and edited the manuscript. All authors read and approved the final manuscript.

## Authors’ information

FO is a postdoctoral research associate in AN’s group and has a strong background in cell biology and bioinformatics. He designed the research, identified CP genes, and analyzed the transcriptome. YL is a graduate student in CG’s group with training in computer science. He performed all RNA-Seq data processing and several bioinformatics studies. XZ is a graduate student in AN’s group studying palatal signaling. CG (Associate professor) has an interdisciplinary background both in molecular and computational biology. He has published a number of computational prediction methods on protein subcellular localization since 2004. AN (Associate professor) is a craniofacial developmental biologist and has extensive experience in cell signaling that governs palate development in TGFβ3 mice.

## Supplementary Material

Additional file 1 **TGFβ3 protein expression in the alleles of TGFβ3 knockout mice.** Western blot analysis show that while homozygous (HM; −/−) palates did not show any expression TGFβ3 protein in the palate collected at E15.5 heterozygous (HT; +/−) palates expressed limited levels of the TGFβ3 protein while the wild-type (WT; +/+) palates expressed higher level of TGFβ3 protein. Click here for file

Additional file 2 Table listing RNA integrity numbers (RIN) to monitor quality and degradation of RNA samples. Click here for file

Additional file 3 Table showing the number of reads per replicate of RNA samples. Click here for file

Additional file 4 **Validation of biological and technical replicates using CummeRbund.** The read numbers per transcript between the biological replicates were compared. Since the read number per transcript (FPKM value) ranged from 0 to >10,000, the read number + 1 was transformed by log10. Each dot represents data from one transcript. There was a high correlation between the two sequencing results (r = 0.98, Pearson’s correlation; p < 0.001 two tail). Click here for file

Additional file 5 **Venn diagrams of all significantly differentially expressed genes.** Differential expression of transcripts within TGFβ3 alleles (TGFΒ3−/−, TGFΒ3+/−, and WT) were compared using Venn diagrams. Lists of differentially expressed genes with their EntrezIDs were uploaded to the R VennDiagram package [75], the query was run, and the detected numbers and gene lists were exported to MS Excel. Relationships among the lists of differentially expressed genes within TGFβ3 allelic mice are depicted as intersections or uniqueness and grouped with their corresponding gestational age (E14.5, E15.5, and E16.5). A significant overlap of differentially expressed genes was observed among the allelic variants at each gestational age. Venn diagrams were not drawn per scale. Click here for file

Additional file 6 Table listing the names of the genes significantly increased from E14.5 to E15.5 followed by downregulation in E16.5 and their. Click here for file

Additional file 7 **Venn diagram of cleft palate (CP) related genes acquired from OMIM and MGI Using the OMIM (Online Mendelian Inheritance in Man) database, we identified 280 genes and loci involved in genetic diseases associated with CP syndromes in humans.** Several studies have developed knockout mouse models which exhibit CP [77]. We used the Mouse Genome Informatics (MGI) international database resource for laboratory mice to identify 128 genes (out of 223 genotypes) that cause CP in mice. The mouse genes were filtered based on gene names only, because some of the same genes were either studied by different labs or have splice variants, and as a result they were listed several times as genotypes in the MGI database. The human and mouse CP-associated genes were compared to find common genes, and the Homologene tool of NCBI [78] was used to compare and detect the orthologs of human and mouse CP related genes. We determined that 50 (out of 280) human CP related genes and loci were not represented, i.e. non-orthologs, within the mouse genome. Click here for file

Additional file 8 **Ideogram of CP genes on chromosomes.** A zoomable Circos image is drawn based on circular composition of CP genes on chromosomes. Data tracks appear inside and/or outside the circular layout. When the PDF file is magnified, tracks include FPKM values of CP genes as histograms and labeled from the outer circle to inner circle as “gene name, 14.5HM, 15.5HM, 15.5HM, 14.5HT, 15.5HT, 16.5HT, 14.5WT, 15.5WT, and 16.5WT”. Click here for file

Additional file 9 Table listing the all the CP genes sorted according to their pattern. Click here for file

Additional file 10 Table showing differentially expressed molecules clustered into GO of cellular biofunctions. Click here for file

Additional file 11 **Stages of palatogenesis in the mouse.** (A) Time-course of palate development in mice. (B-F) Scanning electron micrographs showing oral views of the secondary palate at representative developmental stages [reprinted from Kaufman (Kaufman, 1992) with permission]. Orange lines mark sites of fusion between the medial nasal processes and maxillary processes, white arrowheads point to initial outgrowths of the primary palate, white arrows point to the initial outgrowth of the secondary palatal shelves, red arrowheads mark the initial site of palatal adhesion and fusion, and the yellow arrowhead points to the gap between the primary and secondary palates that will disappear following fusion between these tissues. (G-U) Representative histological frontal sections from anterior (G-K), middle (L-P), and posterior (Q-U) regions of the developing palate at each indicated stage. The middle palate region is flanked by the developing upper molar tooth germs (black arrows in M-P) and corresponds to the palatine region of the future hard palate. The posterior palate region corresponds to the future soft palate. At E11.5 (G,L,Q), the palatal shelf outgrowths arise from the oral surface of the maxillary processes. At E13.5 (H,M,R), the palatal shelves exhibit distinct shapes along the AP axis. By E14.5 (I,N,S), the palatal shelves have elevated to the horizontal position. At ~ E15.0 (J,O,T), the palatal shelves make contact at the midline and initiate fusion by formation of the midline epithelial seam (MES) in the mid-anterior region (arrowhead in O). By E15.5 (K,P,U), palatal shelf fusion is evident in the middle and posterior regions, with complete removal of the MES (black arrowheads in P,U). Remnants of the MES can still be seen in the anterior region (K) at this stage and the palatal shelves also fuse superiorly with the nasal septum. Magnification is not equivalent between stages. MdbP, mandibular process; MNP, medial nasal process; MxP, maxillary process; NS, nasal septum; PP, primary palate; PS, palatal shelf; SP, secondary palate; T, tongue. Adapted from *Bush J O and Jiang R. Development 139, 231–243 (2012).* Click here for file
