# Supplementary figures and images for: Systematic analysis of palatal transcriptome to identify cleft palate genes within TGFβ3-knockout mice alleles: RNA-Seq analysis of TGFβ3 Mice
Source: BMC Genomics. 2013 Feb 20;14:113. doi: 10.1186/1471-2164-14-113 (PMC3618314; doi:10.1186/1471-2164-14-113)

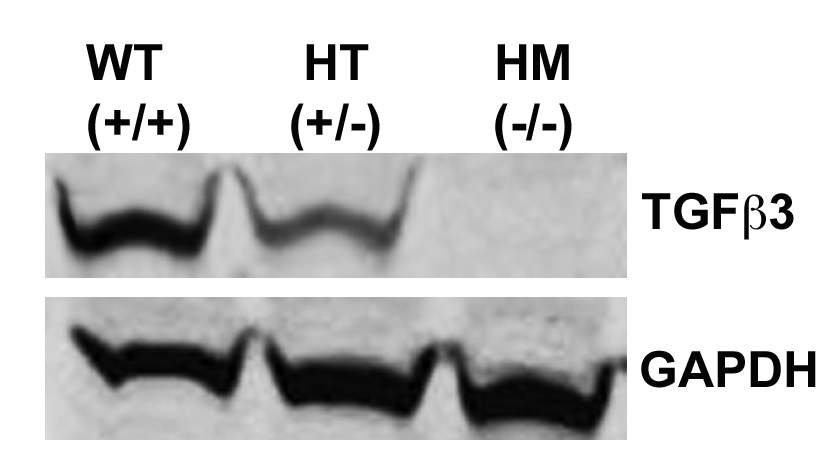

Supplement: Additional file 1 — TGFβ3 protein expression in the alleles of TGFβ3 knockout mice. Western blot analysis show that while homozygous (HM; −/−) palates did not show any expression TGFβ3 protein in the palate collected at E15.5 heterozygous (HT; +/−) palates expressed limited levels of the TGFβ3 protein while the wild-type (WT; +/+) palates expressed higher level of TGFβ3 protein. [file 1471-2164-14-113-S1.tiff]

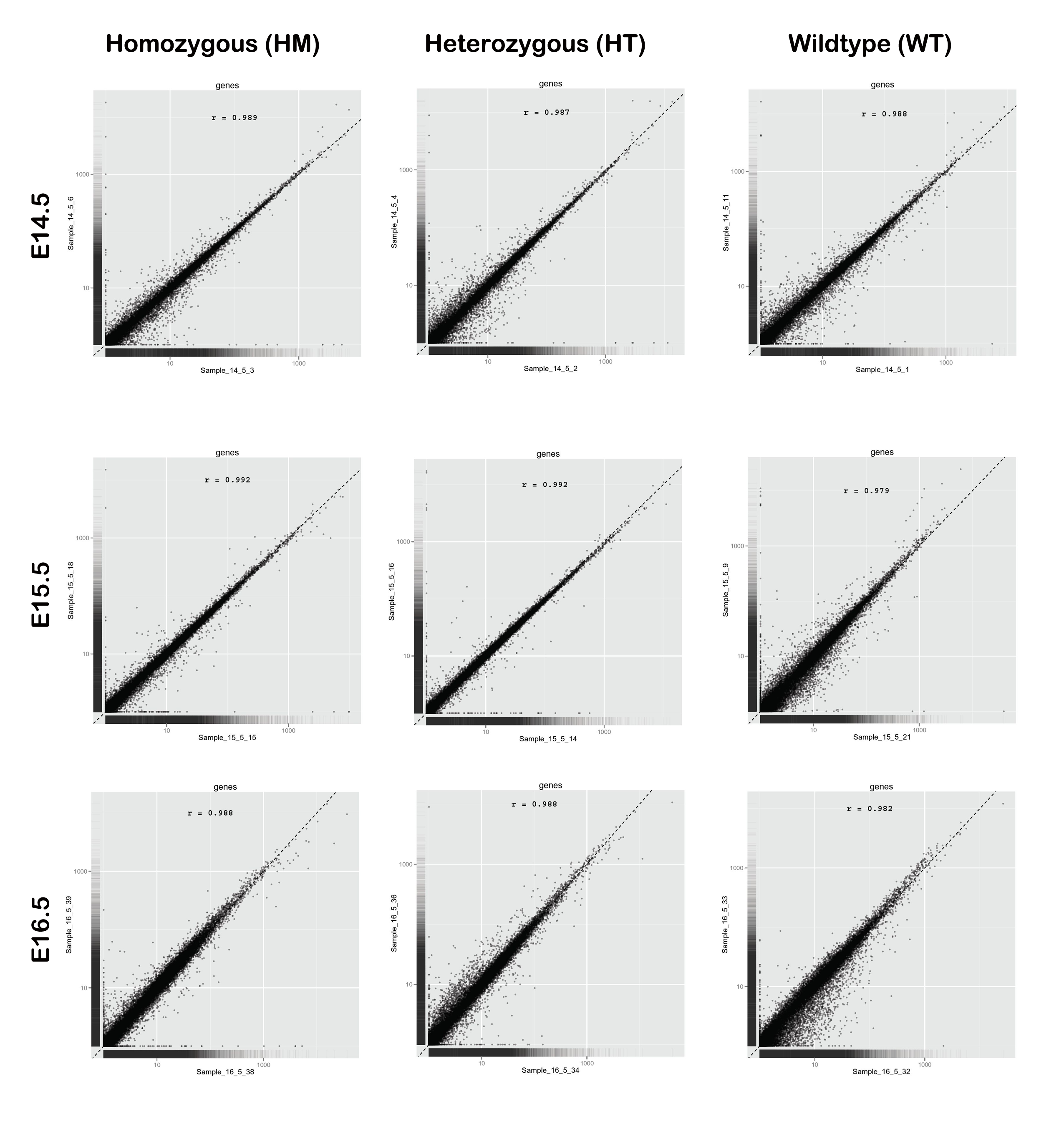

Supplement: Additional file 4 — Validation of biological and technical replicates using CummeRbund. The read numbers per transcript between the biological replicates were compared. Since the read number per transcript (FPKM value) ranged from 0 to >10,000, the read number + 1 was transformed by log10. Each dot represents data from one transcript. There was a high correlation between the two sequencing results (r = 0.98, Pearson’s correlation; p < 0.001 two tail). [file 1471-2164-14-113-S4.tiff]

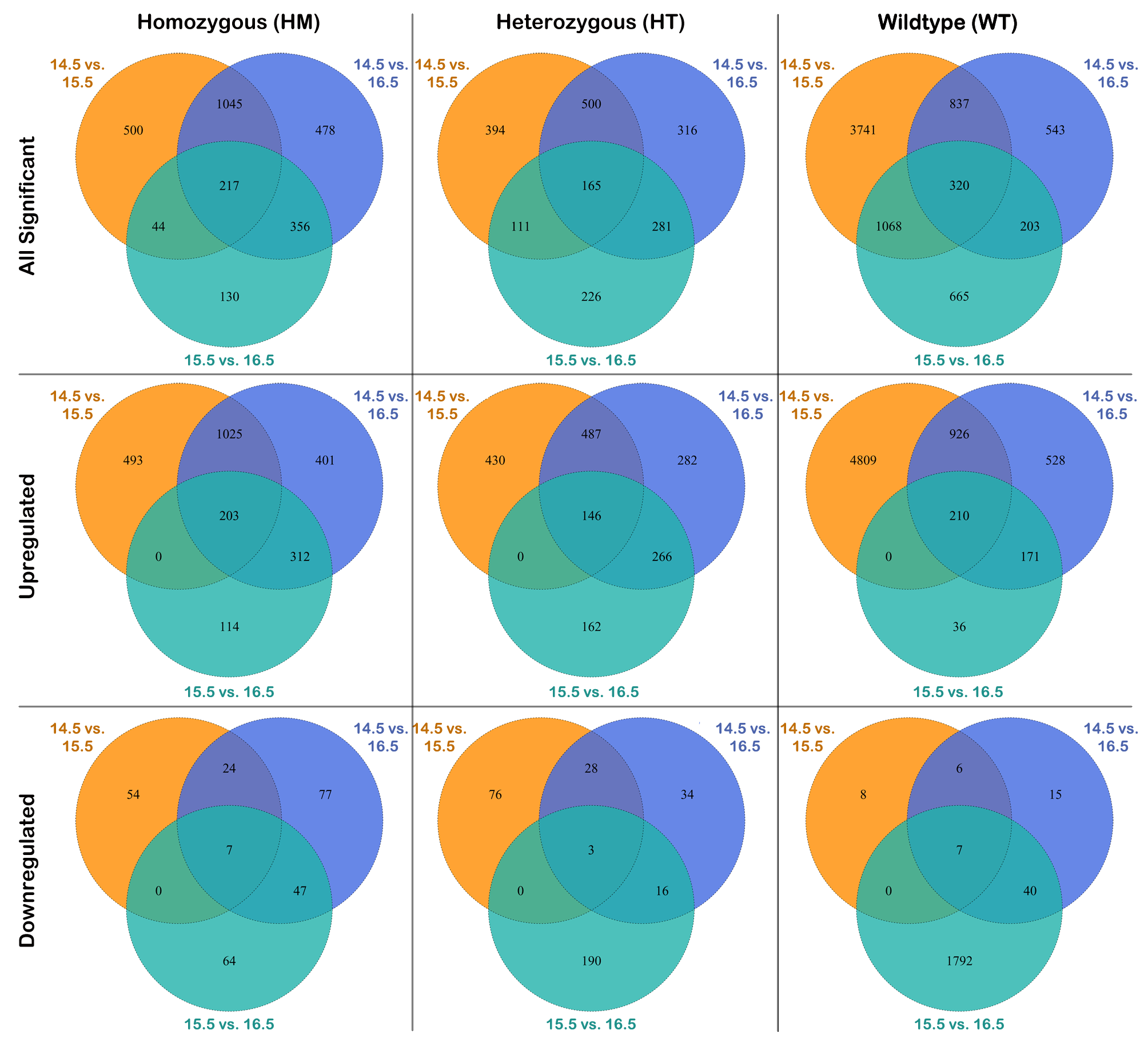

Supplement: Additional file 5 — Venn diagrams of all significantly differentially expressed genes. Differential expression of transcripts within TGFβ3 alleles (TGFΒ3−/−, TGFΒ3+/−, and WT) were compared using Venn diagrams. Lists of differentially expressed genes with their EntrezIDs were uploaded to the R VennDiagram package [75], the query was run, and the detected numbers and gene lists were exported to MS Excel. Relationships among the lists of differentially expressed genes within TGFβ3 allelic mice are depicted as intersections or uniqueness and grouped with their corresponding gestational age (E14.5, E15.5, and E16.5). A significant overlap of differentially expressed genes was observed among the allelic variants at each gestational age. Venn diagrams were not drawn per scale. [file 1471-2164-14-113-S5.tiff]

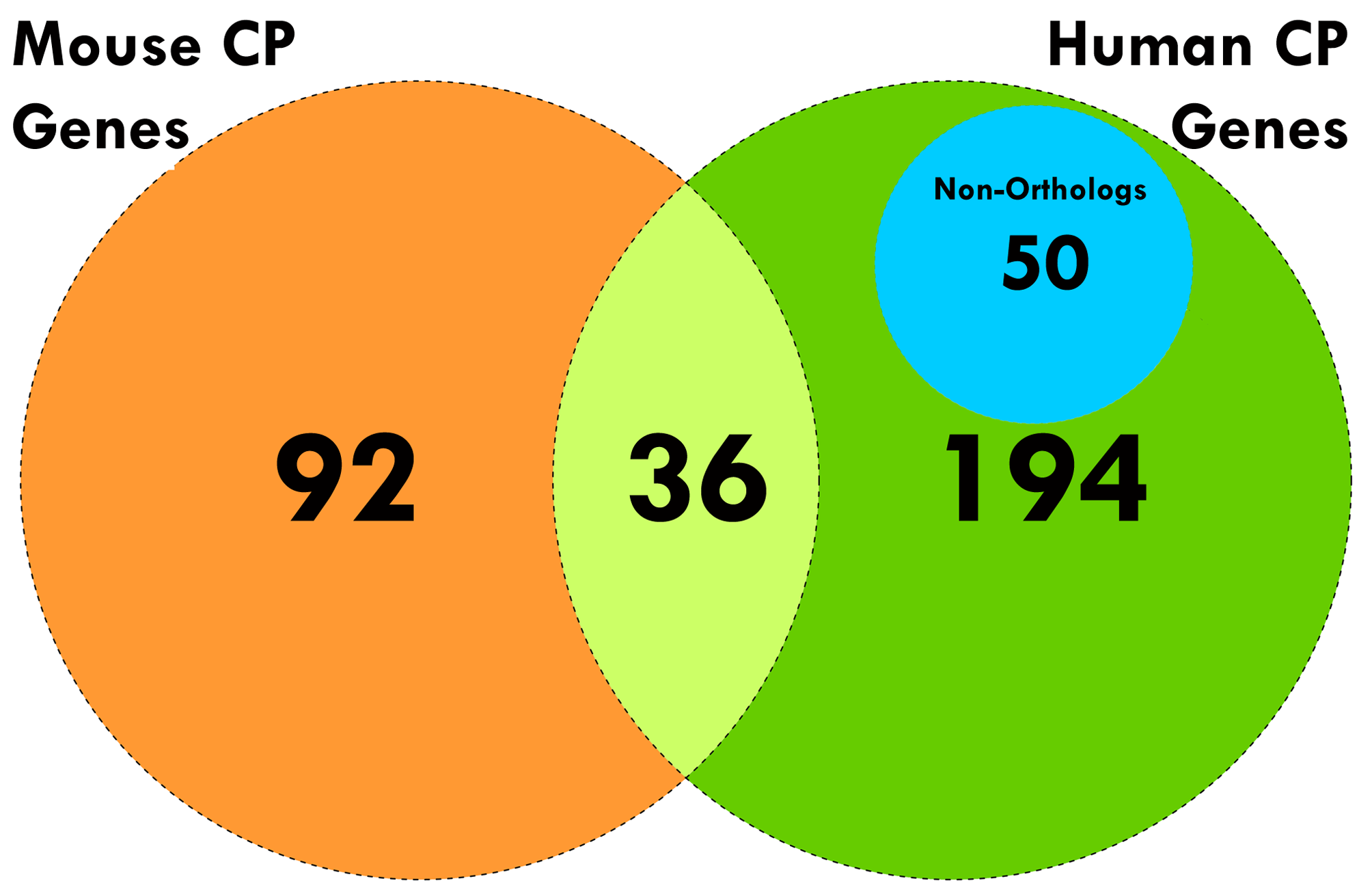

Supplement: Additional file 7 — Venn diagram of cleft palate (CP) related genes acquired from OMIM and MGI Using the OMIM (Online Mendelian Inheritance in Man) database, we identified 280 genes and loci involved in genetic diseases associated with CP syndromes in humans. Several studies have developed knockout mouse models which exhibit CP [77]. We used the Mouse Genome Informatics (MGI) international database resource for laboratory mice to identify 128 genes (out of 223 genotypes) that cause CP in mice. The mouse genes were filtered based on gene names only, because some of the same genes were either studied by different labs or have splice variants, and as a result they were listed several times as genotypes in the MGI database. The human and mouse CP-associated genes were compared to find common genes, and the Homologene tool of NCBI [78] was used to compare and detect the orthologs of human and mouse CP related genes. We determined that 50 (out of 280) human CP related genes and loci were not represented, i.e. non-orthologs, within the mouse genome. [file 1471-2164-14-113-S7.tiff]

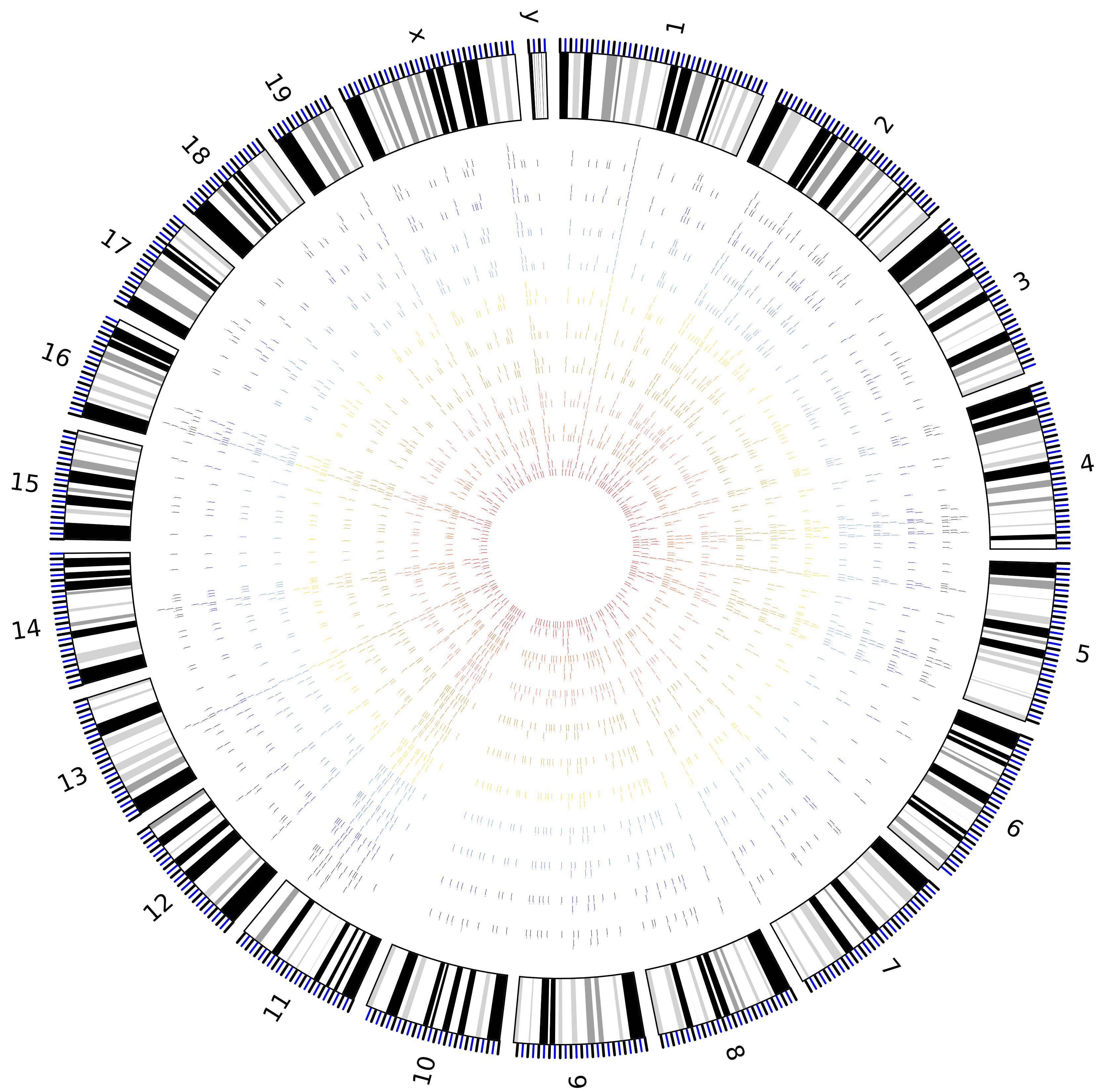

Supplement: Additional file 8 — Ideogram of CP genes on chromosomes. A zoomable Circos image is drawn based on circular composition of CP genes on chromosomes. Data tracks appear inside and/or outside the circular layout. When the PDF file is magnified, tracks include FPKM values of CP genes as histograms and labeled from the outer circle to inner circle as “gene name, 14.5HM, 15.5HM, 15.5HM, 14.5HT, 15.5HT, 16.5HT, 14.5WT, 15.5WT, and 16.5WT”. [file 1471-2164-14-113-S8.pdf]

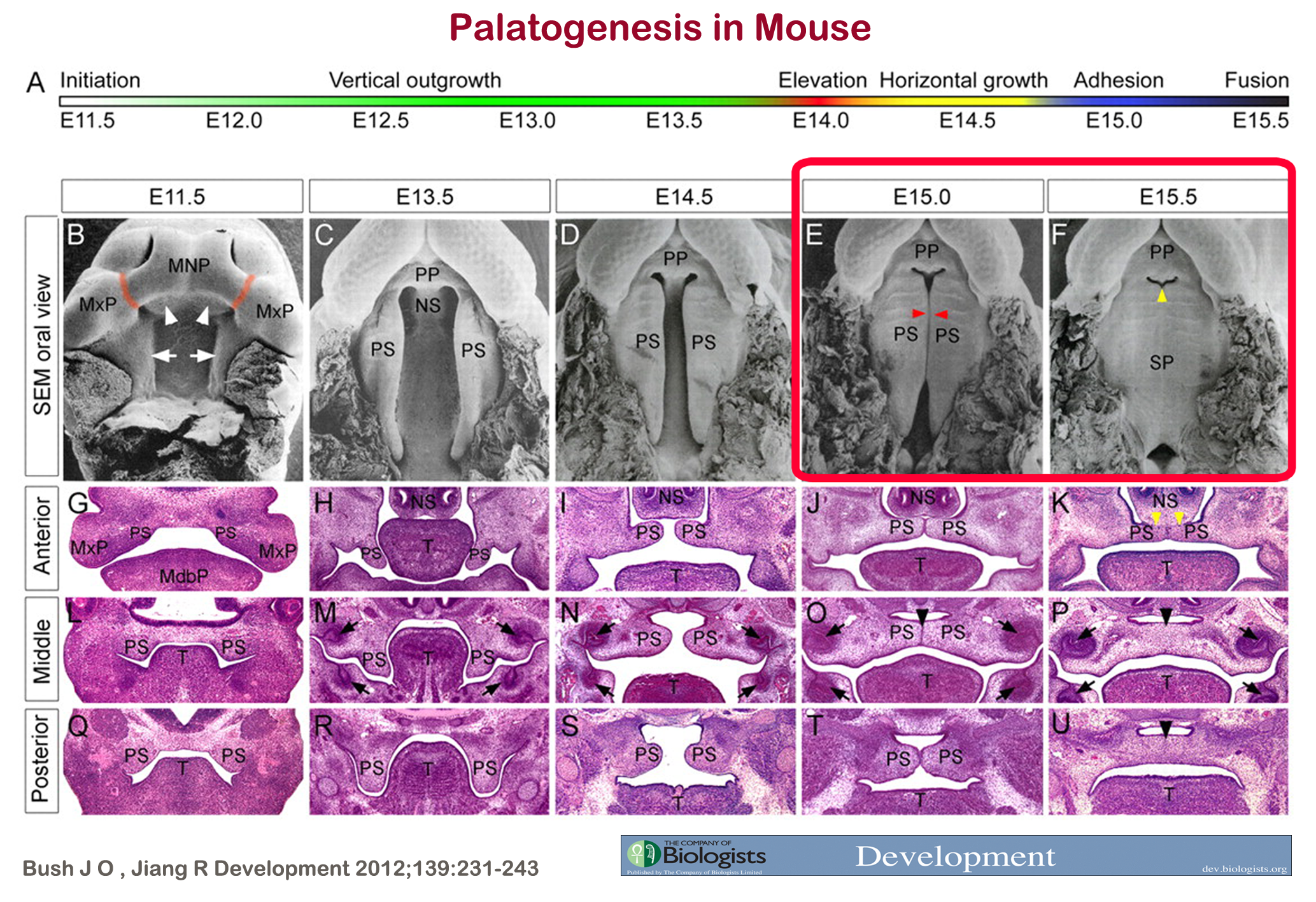

Supplement: Additional file 11 — Stages of palatogenesis in the mouse. (A) Time-course of palate development in mice. (B-F) Scanning electron micrographs showing oral views of the secondary palate at representative developmental stages [reprinted from Kaufman (Kaufman, 1992) with permission]. Orange lines mark sites of fusion between the medial nasal processes and maxillary processes, white arrowheads point to initial outgrowths of the primary palate, white arrows point to the initial outgrowth of the secondary palatal shelves, red arrowheads mark the initial site of palatal adhesion and fusion, and the yellow arrowhead points to the gap between the primary and secondary palates that will disappear following fusion between these tissues. (G-U) Representative histological frontal sections from anterior (G-K), middle (L-P), and posterior (Q-U) regions of the developing palate at each indicated stage. The middle palate region is flanked by the developing upper molar tooth germs (black arrows in M-P) and corresponds to the palatine region of the future hard palate. The posterior palate region corresponds to the future soft palate. At E11.5 (G,L,Q), the palatal shelf outgrowths arise from the oral surface of the maxillary processes. At E13.5 (H,M,R), the palatal shelves exhibit distinct shapes along the AP axis. By E14.5 (I,N,S), the palatal shelves have elevated to the horizontal position. At ~ E15.0 (J,O,T), the palatal shelves make contact at the midline and initiate fusion by formation of the midline epithelial seam (MES) in the mid-anterior region (arrowhead in O). By E15.5 (K,P,U), palatal shelf fusion is evident in the middle and posterior regions, with complete removal of the MES (black arrowheads in P,U). Remnants of the MES can still be seen in the anterior region (K) at this stage and the palatal shelves also fuse superiorly with the nasal septum. Magnification is not equivalent between stages. MdbP, mandibular process; MNP, medial nasal process; MxP, maxillary process; NS, nasal septum; PP, [file 1471-2164-14-113-S11.tiff]
